# Coupled Micromachined Magnetic Resonators for Microwave Signal Processing

**DOI:** 10.3390/mi15020259

**Published:** 2024-02-10

**Authors:** Romolo Marcelli, Andrea Lucibello, Emanuela Proietti, Takuro Koike

**Affiliations:** 1 Institute for Microelectronics and Microsystems (CNR-IMM), 00133 Rome, Italy; andrea.lucibello@leonardocompany.com (A.L.); emanuela.proietti@cnr.it (E.P.); 2 Department of Electronic Engineering, Tamagawa University, Machida, Tokyo 194-8610, Japan

**Keywords:** micromachining, magnetostatic waves, resonators, microwave filters

## Abstract

In this paper, the theory, micromachining technology, and experimental results of the coupling of integrated magnetic film-based resonators for microwave signal filtering are presented. This is an extended contribution to the field of magnetostatic wave coupled resonators, including details about the technological results, circuit theory, and perspective applications for tunable integrated coupled magnetic resonators. An analytical approach using the magnetostatic wave approximation is used to derive the coupling coefficient between adjacent resonators coupled by the electromagnetic field decaying outside the resonators. Then, micromachining employing hot phosphoric acid etching is presented to manufacture integrated coupled resonators. Finally, circuit modeling and experimental results obtained using the ferromagnetic resonance technique are discussed.

## 1. Introduction

Coupled resonators are powerful devices for microwave signal processing when an in-band flat response and a good signal rejection are required for narrowband filtering. A critical coupling between adjacent resonators, corresponding to an electrically matched configuration, is generally required [1,2]. Magnetostatic wave (MSW) filters are microwave tuneable devices, well known in the classic literature about microwave magnetics, used for microwave signal processing based on magnetic garnets in bulk or film structures magnetically saturated with an external DC magnetic field and excited at high frequencies through microstrip or coplanar waveguide circuits [3,4,5,6,7,8]. Linear and non-linear propagation is allowed depending on the excitation power of the high-frequency signal [9,10,11,12], including the chaotic excitation of the spin system [13,14].

Recent advances in microwave magnetics encompass novel materials and nanostructures for miniaturized magnetically tuneable devices in the microwave range and for magnonic and spintronic applications [15,16,17,18,19].

The magnetic garnet films are epitaxially grown materials from molten high-temperature supersaturated solutions [20,21,22,23,24,25], and they are suitable to be used in planar micro-processed configurations involving a few coupled straight-edge resonators (SERs) [26,27]. The best material used for microwave applications is the yttrium iron garnet (YIG), epitaxially grown on gadolinium gallium garnet (GGG) substrates, matching the lattice parameter of the material to be grown by a heterogeneous nucleation mechanism. In principle, the SERs can be connected in a series or organized in a matrix for multi-pole filtering, thus presenting in-line or 90-degree input/output transducers. Using the bulk solution, a mature technology has been developed since the 1970s, bringing commercially available configurations with band-stop and band-pass capabilities over a wide frequency range [28,29], even proposing improvements in the classical configurations [30]. Basic studies are still performed on the single spheres, with interesting results on the excitation of volume and magnetic plasmon resonances using the classical filter arrangement with a wire to excite a microwave signal in a sample biased using an external DC magnetic field [31]. The limitations for YIG are mainly due to the increase in the material losses as a function of frequency and the necessity for a DC magnetic field with a value that is too high to drive the device at very high frequencies. For the above reason, the maximum frequency for utilizing YIG spheres can be evaluated as close to 40 GHz. Millimeter wave applications could be pursued with Li-ferrites, which are also suitable for microwave range applications [32]. Additionally, spheres cannot be easily integrated into a subsystem onto the same substrate, and they need to be considered a hybrid solution with a connectorized device (filter or oscillator). Despite the bulk configuration, YIG spheres are still state-of-the-art from the electric performance point of view, with ongoing patents in microwave magnetics to optimize the current configurations [33]. A well-known limitation of magnetic materials is their sensitivity to temperature, sometimes crossing the ambient temperature and requiring a feedback mechanism. Two solutions are used for this reason: (1) a heating and thermal stabilization of the device, and (2) material substitution with Ga-doped YIG, lowering the magnetization and operative frequencies but having a derivative of magnetization that is lower than that of pure YIG. Nevertheless, for multi-pole filtering, it is desirable to simplify the structure, leading to an easier manufacturing process of both the coupled resonator structure and the feeding system.

This paper develops a theoretical treatment for the microwave response of coupled magnetic planar resonators, detailing the outcomes obtained in earlier investigations conducted by the same research team [27] with additional design, technology, and measurement efforts. The actual realization of integrated series or matrix arrangements is demanded by the utilization of wet etching (based on hot solutions of H_3_PO_4_), starting from an epitaxial yttrium iron garnet (YIG) magnetic film, whose surface is shaped through a photolithographic process and finally selectively etched to get the multi-resonant structure. The result is an integrated configuration composed of coupled resonators, like in [27]. In-band response with maximally flat band shape and very low insertion losses (IL) can be predicted for band-pass filtering applications by adequately tailoring the coupling coefficients of the structure, strictly dependent on the material properties, the geometry, and the RF feeding. The coupling between individual straight-edge resonators (SERs) has been fully modeled in terms of the binding energy of the individual SERs within the MSW approximation. Coupled SERs have been studied, and ferromagnetic resonance (FMR) measurements have been compared with a circuital description of the exploited structure derived using the MSW theory. An insertion loss of IL < 3 dB at the X-band has been predicted. As a result, a fully planar structure aims to replace the classical multi-pole YIG sphere-based devices by utilizing a micro-machined structure for in-line (in the case of series resonators) and 90° input/output (for matrix configuration) filters. The micromachining technique of coupled resonators is described in detail, and preliminary experimental results on actual structures are presented and discussed. Finally, a circuital approach is proposed, and predictions are given for potential utilization in device structures. Our group began the original study on coupled MSW resonators by initially focusing on isolated single resonators to verify the coupling through FMR experiments. Then, the micromachining technology was studied, and after obtaining reasonable results on the surface quality of the etched samples, the theory was completed to describe the interaction between resonators, confirmed by new FMR measurements. In the end, the circuital approach was developed to explore the expectations of the integrated structure using electrically matched conditions at the input/output ports of the entire configuration. So far, in our paper, we outline the contribution of at least four activities, two of them experimental:

- Technology for manufacturing coupled planar MSW resonators.
- FMR measurements on series and matrix-arranged resonators.
- Full theory of the coupling for the investigated structures.
- Expected circuital performance assuming electrical matching of the resonator configurations.

## 2. Theory of Coupled Magnetostatic Wave Resonators

Magnetostatic waves (MSWs) have been studied and analytically treated since the 1950s and have been considered for linear and nonlinear excitation in continuous wave and pulsed regimes. The MSW approximation leads to the Polder tensor, linearly relating the high-frequency magnetization vector m in the magnetic spin system and the microwave field h [34]. From the Maxwell equations and in the limit for a purely dielectric material, we have ∇×h=0, and we can write h=−∇ψ (see, for instance, [5]). Using this approximation, the Walker equation is originated, with solutions for the wavefunction ψ as plane waves propagating in the magnetic medium or causing a resonance condition with the wavevector k determined by the finite size of the sample. In principle, a resonator should allow resonance modes limited to the sample, but the edges of the resonator are free and not limited by metal boundaries; then, some energy is lost laterally, and leakage is responsible for the coupling between adjacent resonators.

Magnetostatic wave (MSW) planar resonators are epitaxially grown yttrium iron garnet (YIG) magnetic films, absorbing power in the microwave range. They usually have a rectangular shape and are defined as straight-edge resonators (SERs) for narrow-band high-quality-factor filters or in the feedback sections of microwave tunable, low-phase-noise oscillators. Band-pass SERs are placed between two microstrip transducers with side coupling for the device’s input and output. The size is chosen to fulfill the conditions of electrical matching and frequency selectivity of the resonator itself. Micromachined configurations of the microstrip transducers have been recently proposed for optimizing electrical matching, with advantages in the insertion losses and the bandwidth enlargement [35,36]. The single MSW SERs and their mutual coupling have already been studied in [26,37,38], and an interpretation of the coupling in terms of the magnetostatic wave potential has been given. The present contribution fully formalizes the problem, and the technology for obtaining integrated, micromachined structures has been adopted for coupled rectangular SERs in series and square SERs organized in a matrix.

The coupling can be described as performing the overlap between the magnetostatic potentials ψ. Each SER is coupled with the first neighbor (on the same plane) by the overlap of the EM fields originating from the MSW potential decaying at the interface between the sample and air on the top surface and the bottom one of a magnetic film along directions orthogonal to the surface. All the literature about MSW resonators is based on the excitation of modes confined in the (*x*, *y*)-plane of the sample, but with an exponential decay of the MSW potential only in the *z*-direction, normal to the film plane. In the region of separation between two SERs, an additional exponential decay of the MSW potential can reasonably be assumed along the (*x*, *y*)-directions, i.e., parallel to the film plane and orthogonal to the sample edges. In what follows, we shall assume that the coupling will be determined mainly by the *x*-component of the MSW potential or the *y*-component for a right-angle coupling, separating the contributions of the wave-vector components for in-line and 90° coupling.

**Figure 1:**
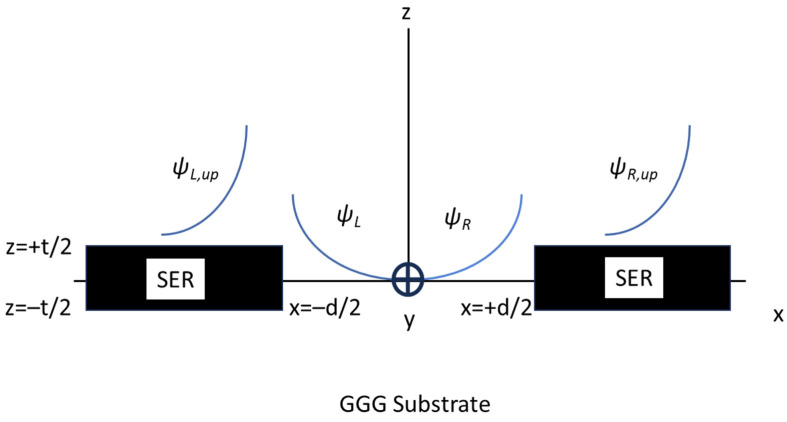
Schematic representation of the coupled SERs considering the relative position and the decay of the wave function outside the samples. The left (*L*) and right (*R*) wave functions decay exponentially from the SER side, and the same happens for the z-contribution, identified here by the (*L,R-up* indexes). For the sake of simplicity, only the upper part of *ψ* is shown, but the contribution on the lower part is symmetric because the substrate is not ferromagnetic.

**Figure 2:**
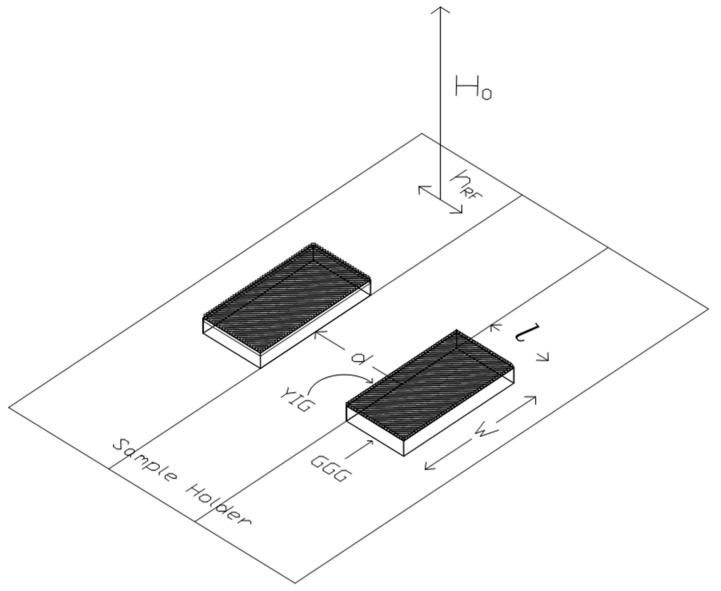
Two SERs, obtained by cutting a YIG film (black in the figure) epitaxially grown on a GGG substrate (white in the figure) with planar size W = width and *l* = length, are separated by a distance *d*. A DC magnetic field H_0_ normal to the magnetic medium is imposed to saturate the magnetic medium’s spin system, allowing for an orientation of the static magnetization almost parallel to H_0_. In contrast, an RF magnetic field h_RF_ at frequencies in the microwave range is excited in the plane of the film.

The trend of the magnetostatic wave potentials outside each sample, along the *x*-direction, is given by the following:

(1) $$\psi_{L,R}=\psi_{0}e^{\mp k_{x}\left(x\pm \frac{d}{2}\right)}$$

where *L* = left and *R* = right, assuming that the coupled SERs are placed in a frame with the origin centered to the coupling edges, separated by a distance *d*. *k_x_* is the in-plane component of the wave vector excited in the resonator along the *x*-direction. The schematic diagram for the coupling between the MSW wave functions is shown in Figure 1. At the same time, the general arrangement of the individual coupled resonators is given in Figure 2 elaborated here for the reader’s convenience from [26].

The complete potential outside of the resonator accounting for both the contributions in Equation (1) and the *z*-component is given by:

(2) $$\psi =\psi_{0}\left[e^{-k_{x}\left(x+\frac{d}{2}\right)}+e^{k_{x}\left(x-\frac{d}{2}\right)}\right]e^{-k_{x}\left(z-\frac{t}{2}\right)}$$

where *t* is the thickness of the film, and the exponential decay outside the magnetic film is due to the imaginary solution for the Walker equation in the MSW approximation.

Assuming *x*- and *z*-only dependence for the MSW excitation, it turns out that *μk_x_*^2^ + *k_z_*^2^ = 0. In the case of air propagation, the decay is obtained by the imaginary solution for *μ* = 1, which corresponds to *k_z_* = *ik_x_*.

The magnetic energy *E_M_* exchanged between two identical resonators in the intermediate region of air between them can be obtained from the classical volume integral definition:

(3) $$E_{M}=\frac{1}{2}\int {\left|h\right|}^{2}dV$$

where the integration volume *V* is defined in the plane (*x*, *y*) by the width *W* of the SER and by the distance *d* between the two SER edges, while *z* ranges between *t*/2 and +∞ when *z* > 0, or −∞ and −*t*/2 when *z* < 0 (like in Figure 1).

The internal energy does not contribute to the coupling between resonators but to the resonance of the individual SERs.

h is the superposition between the microwave fields outside the SER edges. The *x*- and *z*-components in the MSW approximation are obtained from:

(4) $$h_{x}=\frac{\partial \psi }{\partial x}=2k_{x}\psi_{0}e^{-k_{x}\frac{d}{2}}sinh(k_{x}x)e^{-k_{x}\left(z-\frac{t}{2}\right)}\;h_{z}=\frac{\partial \psi }{\partial z}=-2k_{x}\psi_{0}e^{-k_{x}\frac{d}{2}}cosh(k_{x}x)e^{-k_{x}\left(z-\frac{t}{2}\right)}$$

By using the above definitions for the ***h***-components, we have:

(5) $$\int |h{|}^{2}dV=4|\psi_{0}{|}^{2}k_{x}^{2}e^{-k_{x}d}W\cdot 2\int_{\frac{t}{2}}^{\infty }e^{-2k_{x}\left(z-\frac{t}{2}\right)}dz\cdot \int_{-\frac{d}{2}}^{\frac{d}{2}}\left[1+2{sinh}^{2}(k_{x}x)\right]dx$$

The integration is performed between the two SERs, where the two scalar potentials overlap. *W* is the side of the SER orthogonal to the side for which we are calculating the coupling, and *t* is the thickness. The factor 2 in front of the *z*-integral is because contributions equal between them are obtained from the integral for *z* < −*t*/2 and *z* > *t*/2.

From Equation (5) we obtain:

(6) $$E_{M}=|\psi_{0}{|}^{2}W\cdot F\left(d\right)\;F\left(d\right)=k_{x}d\cdot e^{-k_{x}d}\left[1+2\frac{sinh(k_{x}d)}{k_{x}d}\right]$$

When the two resonators are far from each other, the function *F*(*d*) is unitary, while it vanishes for *d* = 0. Since there must be no effective coupling when the two SERs are distant between them, the coupling energy can be written as:

(7) $$E_{coupling}=|\psi_{0}{|}^{2}W\left[1-F\left(d\right)\right]$$

Using this assumption, *E_coupling_* = 0 when the two resonators are at *d* = 0, because this condition defines a new SER with a doubled side. On the other hand, the energy of a single SER can be written as:

(8) $$E_{SER}=\frac{1}{2}\chi \prime \prime \int_{SER}|h{|}^{2}dV=\frac{1}{2}\chi \prime \prime k_{x}^{2}|\psi_{0}{|}^{2}W\cdot l\cdot t$$

The volume *V* is now that of the SER. Non-decaying plane waves describe the sample’s resonance condition so that the imaginary contributions are simplified when ${\left|h\right|}^{2}$ is evaluated. *χ*″ is the imaginary part of the Polder susceptibility tensor, calculated by a linear approximation of the microwave components of the magnetization using the equation of motion around a static DC magnetic field. At resonance, when the radian frequency of the RF field exciting the sample is equal to the natural frequency of the spin system, it will be *χ*″ = (1/2)(4*πM_S_*/Δ*H*), where 4*πM_S_* is the saturation magnetization of the sample, and Δ*H* is the magnetic full linewidth measured through an FMR experiment. This is a reasonable approximation, even if the frequency of resonance for the uniform mode differs from the first excited mode in a finite sample. It might be outlined that the analytical treatment developed in this paper follows the usual approximation of a pinned spin along the edges of the magnetic film. This is an ideal case, but in practical conditions, the edges are not ideal, and sometimes phenomenological corrections are performed, introducing a “pinning” factor to justify the disagreement with the experimental findings.

From an electrical engineering point of view, an SER, like any other resonator, can be modeled using an RLC series circuit, where L is the equivalent inductor, C is the capacitor, and *R* is the resistor accounting for the material losses contribution.

The coupling energy for two magnetically coupled resonators can also be written in terms of a coupling inductance *L_coupling_* and the current *I* flowing in each of the two identical resonators, as *E_coupling_* = (1/2)*L_coupling_ I*^2^. Since *L_coupling_* = *KL*, where *K* is the coupling coefficient, it turns out that in our case, *E_coupling_* = *KE_SER_*, where *E_SER_* is the energy of the single resonator.

Concerning the excited wave vectors, they depend on the SER dimensions, and it is generally assumed that *k_x_* = *n_x_*π/*l*, where *n_x_* is an integer odd number, with the mode described by sinusoidal functions having maxima in the center of the sample and vanishing on the sides.

By using the above discussion and the previous definitions, the coupling coefficient between two SERs is obtained by the ratio of the energies in Equations (7) and (8) as:

(9) $$K=\frac{4}{\pi^{2}n_{x}^{2}}\frac{\Delta H}{4\pi M_{S}}\frac{l}{t}\left[1-F\left(d\right)\right]$$

It is worth noting that the coupling factor K depends not only on the distance d between the two resonators but also on the geometry of the individual resonators (*l* and *t*) as well as on their magnetic properties (4*πM_S_* and Δ*H*) and the order of the excited MSW mode. Moreover, high-order modes are depressed by a factor (1/*n_x_*^2^) and should not contribute effectively to the coupling mechanism. Finally, at least within the approximations, such a coupling is independent of the frequency. This is typical for MSW resonators, whose wavevectors are defined by their geometry, even if the *k*-values enter the definition of a dispersion relation linking the wavevector, the frequency, and the magnetic fields (RF and DC). On the other hand, this is an advantage, as the same resonance mode can be moved to another frequency just using the external DC magnetic field.

The coupling inductance between the SERs and the corresponding frequency shift for the entire filter must be predicted to build up an equivalent electrical circuit for the resonating structure. They can be obtained using the theoretical treatment in [1,26,38,39] and from the above discussion.

**Figure 3:**
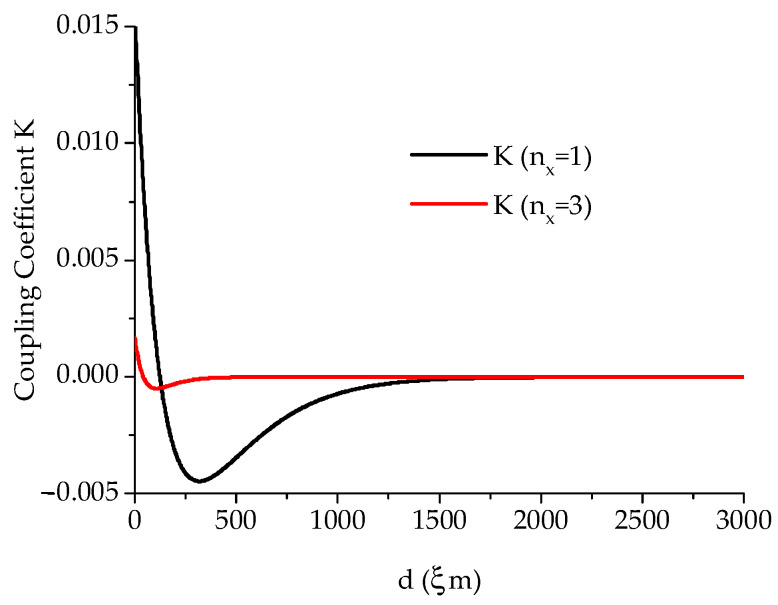
Predicted coupling coefficient *K* for two resonators having *l* = 800 μm and *t* = 30 μm as a function of the distance *d* between them for the two modes *n_x_* = 1 and *n_x_* = 3. The values 4πM_S_ = 1760 gauss and Δ*H* = 3 Oe have also been imposed.

**Figure 4:**
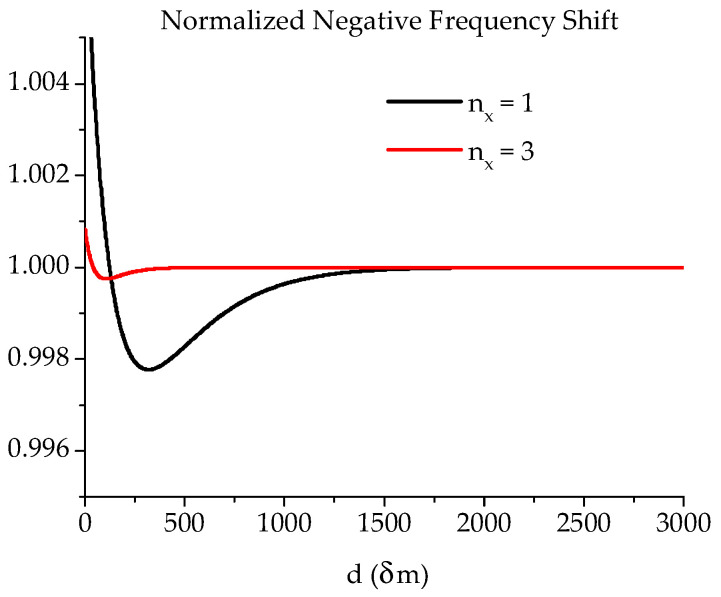
Normalized frequency shift (*ω’_res_*/*ω_res_*) due to the coupling of magnetostatic wave resonators as a function of the distance *d* between them using the same data imposed in Figure 3.

The validity of Equation (9) is limited to its practical utilization for typical dimensions used for magnetostatic wave devices. The limit conditions are fulfilled, being the two SERs uncoupled when the separation is significant (*K* → 0 when *d* → ∞), and they change their identity when the distance between them vanishes (*d* → 0), becoming another SER with doubled dimensions. It means that the resonance frequency will be unchanged for *d* → ∞ compared to that of the single SER, and it will change when the coupling becomes effective. The dependence on the thickness, for which a divergent response is expected when *t* → 0, is more critical. Still, the theory should be corrected because the magneto-exchange contributions must be included for sub-micron films [40]. From the definition of the coupling inductance as *L_coupling_ = KL*, the resonating frequency of the system composed by the two coupled identical series resonators will be given by [1,2,26,37,38], and:

(10) $$L_{coupling}=KL\;\;\frac{\omega_{res}^{\prime }}{\omega_{res}}=\frac{1}{\sqrt{1-K}}$$

where *ω_res_* and *ω*′*_res_* are the radian frequencies of resonance of the individual uncoupled SERs and the coupled structure, respectively, and L is the equivalent lumped inductor for the single SER. The shift corresponds to the frequency location of the ripples of the band-pass filtering response, which can also be used to define the coupling degree between two resonators. So, the central frequency of two inductively coupled resonators (magnetic coupling) will remain approximately the same, but the filter in-band response will be re-shaped according to the discussions in [1,2,26]. The external coupling, which will account for the coupling between the total resonating structure and the external world, is also responsible for shifting the central frequency. Such a shift will vanish as the external coupling coefficient becomes closer to a unitary value. The energy and the frequency shift calculated based on the previous equations are a decaying function of the distance between the two SERs with a binding located at a distance *d* = λ/2 for the minimum but with a critical coupling for *d* = λ/4 to obtain the best electrical performance (flat in-band response), which practically corresponds to a value that is half of the side of the SER along the coupling direction. Because of the necessity to introduce effective dimensions for the SER, an effective wave vector must also be computed due to the non-homogeneity of the internal DC magnetic field due to the de-magnetization factors for the sample [39,41]. It is generally assumed that, with the exception completed for second-order corrections, the effective length is corrected by using *l_eff_* = *l* − 4*t*, and it will result in an effective wave-vector *k_x,eff_* = *n_x_*π/*l_eff_*. This is valid when *l*^2^ << *W*^2^ [14]. As a further refinement, more precise calculations of the internal DC magnetic field distribution allow an exact analytical treatment of the effective dimensions, as in [39,41]. Still, only a higher-order correction is introduced to improve the theory. When the two SERs are face-to-face (*d* = 0), a new resonator is defined with double size on the short side. This will cause the wavevector at resonance for the main, (1,1)-mode to be shifted by the ratio $k_{res}^{\prime }/k_{res}\approx 1/2$, i.e., we shall experience the excitation of lower *k*-values (and lower radian frequencies for the volume MSWs). As an example of the previous discussion, the expected trends of *K* (Equation (9)) and the normalized frequency shift (Equation (10)) for two MSW SERs with *t* = 30 μm and *l* = 800 μm, i.e., with *l_eff_* = *l* − 4*t* = 680 μm and *k_x,eff_* ≈ 46 cm^−1^, are shown in Figure 3 and Figure 4.

For square-shaped resonators, the condition *l^2^* << *W^2^* is, in principle, no longer valid, but also, in the case of the matrix, we shall assume that the side length and its wavevector component dominate the coupling contribution to develop a theory for the matrix which involves one-dimensional considerations extended to a plane made by a ladder network with no diagonal links.

As previously discussed, each resonator will couple with the first neighbor by using the overlap of the EM field that starts to decay from the side of both resonators. Following this approach, each resonator “internal” with respect to an nth-order matrix has four first neighbors, and each resonator “external” will have three of them or just two first neighbors if it is in a corner.

**Figure 5:**
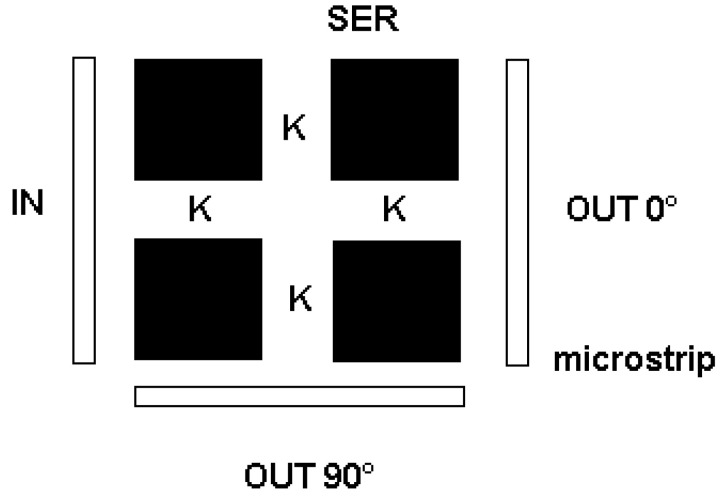
General scheme of a 2 × 2 matrix of SERs to be used with microstrip connection for microwave signal processing. In-line and 90° input/output are proposed. K defines coupling coefficients involving the electrical matching between SERs. For the FMR experiment, the microstrips are substituted by coupling with a TE_102_ cavity.

Different kinds of excitations can be assumed: an electrical transducer with side coupling for the first row (or column) when band-pass characteristics are required or with top coupling with respect to a row (or column) belonging to the matrix of resonators when a band-stop response is needed. Moreover, the transducers used for the excitation can be analogous to the case of the series of resonators, coupled just with the first and last row (or column) of the matrix or involving the entire matrix. In the first case, the signal is transferred from one row to another. In contrast, in the second case, a collective excitation is induced in the structure, as is the case of a ferromagnetic resonance (FMR) experiment. A schematic arrangement of the matrix of resonators is shown in Figure 5.

In the present work, a matrix composed of square SERs having a side *l* = 800 μm long and a thickness *t* = 30 μm has been measured, with a nominal 400 μm of separation between the SERs (λ/4-coupling).

The phase velocity along a microstrip transducer to be used for a device configuration is (*ω*/*k*)_*microstrip*_ = *c*/√*ε* = 3 × 10^10^/√9.8 ≈ 9.6 × 10^9^ cm/sec (the dielectric constant of the alumina substrate *ε* = 9.8 has been used, and not the effective one of the microstrip transmission line, which is lower, so this value is under-estimated). In the case of the MSWs, we have *k_x,eff_* = *n_x_*π/*l_eff_* ≈ π/0.068 cm^−1^ ≈ 46 cm^−1^ and (*ω*/*k*)*_MSW_* ≈ 2π × 9.25 × 10^9^/46 ≈ 1.26 × 10^9^. Concerning the wavelength, we have *λ_microstrip_* = *c*/(*f*√*ε*) = 3 × 10^10^/(9.25 × 10^9^ × √9.8) ≈ 1.04 cm (under-estimated), and *λ_MSW_* = 2*π*/*k_res_* = 2π/46 ≈ 0.13 cm. For *f* = 9.25 GHz, we are close to an order of magnitude in the difference between *λ_microstrip_* and *λ_MSW_*, which should be enough to assume that *λ_microstrip_* >> *λ_MSW_*. Under these conditions, the individual SERs are excited with no phase change along the microstrip signal.

The theory for coupling two SERs based on the MSW approximation does not allow the inclusion of the binding range experimentally detected using an FMR experiment.

The frequency shift calculated based on the previous equations is a decaying function of the distance between the two SERs. The measured frequency shift looks like the binding energy calculated in solid-state physics for the electrostatic interaction between ions based on the contribution of the first neighbors. Then, a fitting function with the same analytical formulation of binding energy has been used for this phenomenological reason.

The general formula can be written as:

(11) $$\frac{\omega_{res}^{\prime }}{\omega_{res}}=1+Aexp\left(-\frac{x}{\rho }\right)-\frac{B}{x}$$

where *A*, *B*, and *ρ* are constants to be determined.

From past experimental results [26,27], the ratio between the two resonance frequencies has a minimum corresponding to x=l, where l is the short side of the SER, to be also used for the computation of the wave vector at resonance *k_res_*.

In the ideal case, it should be l=λ/2, but the correction for the effective value of λ due to de-magnetization contributions is re-absorbed by the approximated definition $k_{res}=\pi /l_{eff}=\pi /\left(l-4t\right)$ [27,28,29,30,31,32,33,39,41]. By reasonably assuming that the “extension” of the interaction can be measured as *ρ =* 1/*k_res_*, the minimum for the ratio is obtained when $B=Ak_{res}lexp\left(-k_{res}l\right)$ and the previous equation is transformed in

(12) $$\frac{\omega_{res}^{\prime }}{\omega_{res}}=1+A\left[exp\left(-k_{res}l\left(l\right)\right)-\frac{k_{res}l\;exp\left(-k_{res}l\right)}{\frac{x}{l}}\right]$$

where now *A* is the only unknown. We performed an FMR experiment using two SERs with planar dimensions l×W = 0.96 × 2.9 mm^2^ and a thickness of 45 μm. From the geometrical dimensions, we can calculate the effective wave vector at resonance as $k\approx \pi /l_{eff}=\pi /\left(l-4t\right)\approx 40\;{\mathrm{cm}}^{-1}$, where $l_{eff}=l-4t$ is the effective length, which accounts for the thickness’s influence on the resonator’s effective planar dimensions. This is valid because $l^{2}<<W^{2}$.

By fitting *A* with the experimental data and using *k_res_* = 40 cm^−1^, i.e., the effective value of the wave vector, it turns out that *A* = 0.06 is the case in our case.

**Figure 6:**
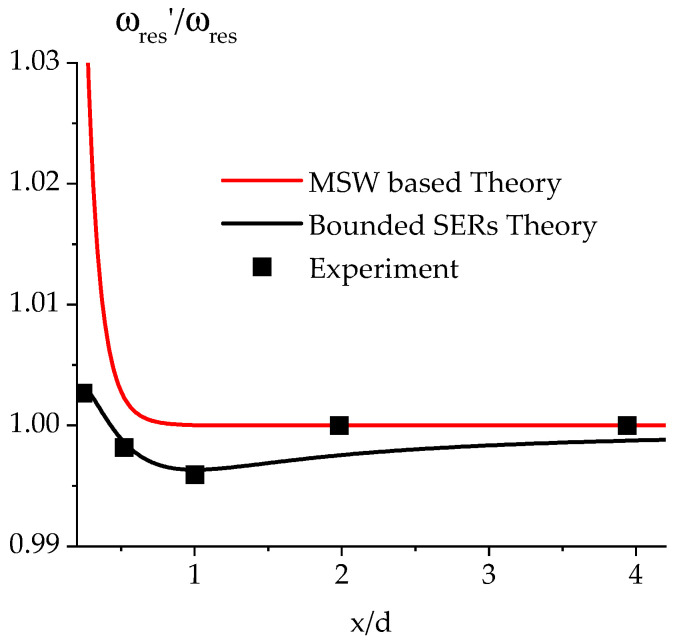
Fit of the experimental data taken from an FMR experiment (bounded SERs theory and experiment) compared with the expectation of the resonance frequency from the MSW theory.

The results are shown in Figure 6.

The previous Figure 6 shows that the coupling based only on the MSW potential is insufficient for predicting the coupling between the two SERs. It can be considered a first-order approximation, but the binding term should substitute it.

As a result, the coupling coefficient based on the coupled SERs theory is:

(13) $$K=1-{\left(\frac{\omega_{res}}{\omega_{res}^{\prime }}\right)}^{2}$$

where the ratio between the two radian frequencies can be obtained from the previously developed “bounded SERs” theory.

In the end, it was possible to have a model of the coupling by studying individual resonators separated in terms of the wavelength or fractions of it.

## 3. Technology

This section presents the technology for manufacturing a structure composed of coupled straight-edge resonators (SERs). Series configurations of rectangular cascaded SERs and square resonators organized in a matrix have been obtained by properly masking the magnetic film to be etched and using a wet chemical etching technique in hot phosphoric acid to obtain the coupled SERs directly onto the garnet film. The etching must be performed down to the GGG substrate hosting the YIG film to separate the magnetic resonators among them (i.e., the structures having magnetic properties sensitive to both the DC magnetic field and the RF microwave field) and to obtain coupled integrated SERs fixed onto the diamagnetic GGG substrate. Chemical etching is not the only solution to manufacture the coupled SER structures. Dry etching can also be used to remove the garnet in the areas not protected against the material removal. To etch the garnet in the case of thin films (up to a few μm) or to obtain simple grooves on the film surface, reactive ion etching (RIE) can also be used. On the other hand, thick films are necessary to obtain a good electrical coupling between the magnetic resonator and the microwave transducer. In this case, the more aggressive chemical etching is preferred, even if specific rules must be followed to get high-quality structures. In particular, the chemical etching is activated as a function of temperature T with a typical Arrhenius-type trend, and it begins to give evidence for a turbulent response when t > 160 °C. For this reason, the suggested range for obtaining good quality etched surfaces is 120 °C < T < 160 °C.

The SERs have been manufactured using a YIG film epitaxially grown on a <111>-oriented (crystallographic direction) double-sided polished GGG substrate, one inch in diameter and 500 μm thick. As well established, GGG is the best solution for the epitaxial growth of YIG, because of the small difference in the lattice constant and no interference with the ferrimagnetic properties of YIG. For the realization of the coupled resonator structures, one photolithographic mask is necessary, and the entire fabrication process is divided into three main steps, as from the following process flow:

1st step—preparation of the GGG substrate (ultra-sonicated in acetone and isopropanol for surface cleaning).
2nd step—thermal evaporation of a thin film of Cr used as an adhesion layer and then of Au. The metallization is made to mask the YIG film and to cover the areas of the garnet that has not to be etched by hot phosphoric acid (H_3_PO_4_ at 140 °C)
3rd step—total immersion of the YIG/GGG wafer into the hot solution of H_3_PO_4_ to etch the areas of the garnet not protected by the Au layer down to the GGG substrate.

**Figure 7:**
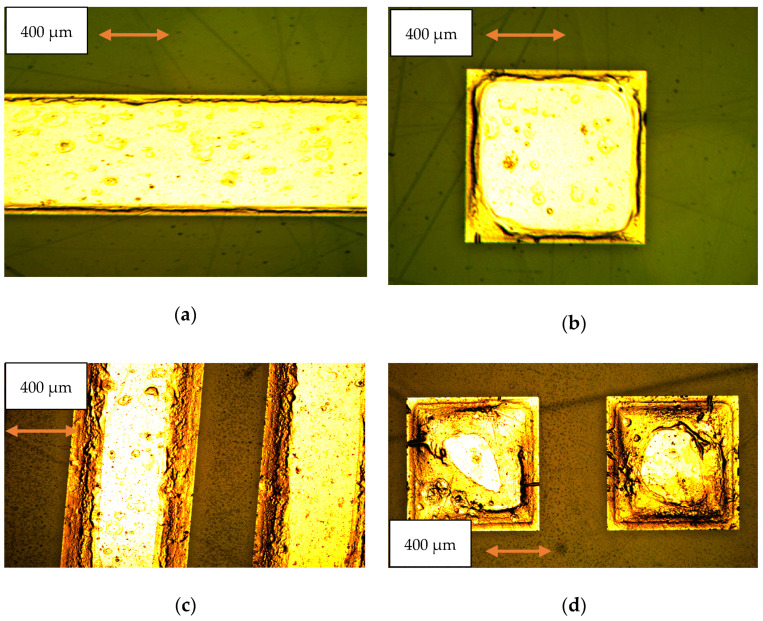
Etched YIG resonators on a GGG substrate. In (**a**,**b**), a detailed view of the rectangular and square resonators is shown after 40 min of etching at 140 °C, while in (**c**,**d**), the result of a 60 min etching is shown by looking at the structures with coupled rectangular and square resonators, respectively. In this case, the single square resonators are initially designed to have an 800 μm edge and a separation of 400 μm, corresponding to λ/2 resonators separated by a λ/4 distance. In comparison, the rectangular ones are 3000 μm long.

**Figure 8:**
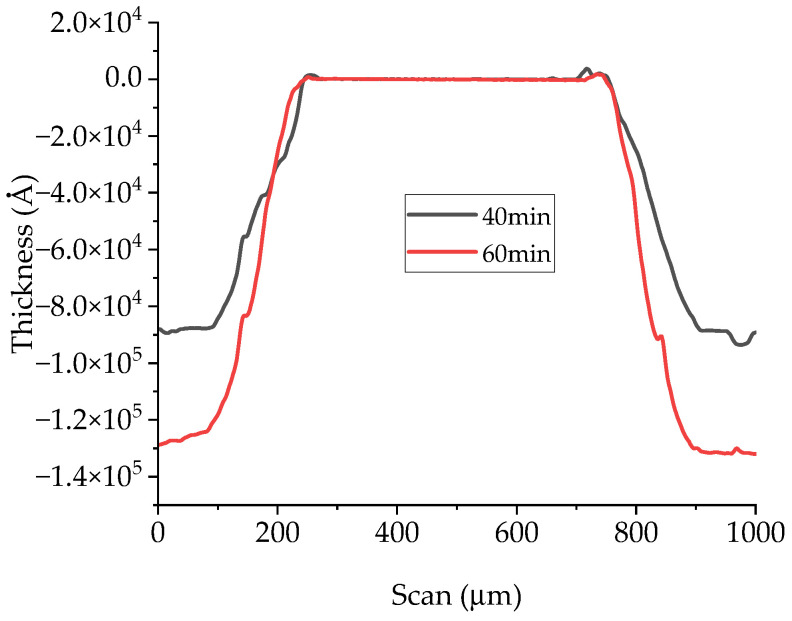
Thickness profile of the etched square resonator during the etching process after 40 min in hot phosphoric acid and after 60 min. A linear slope can reasonably approximate the profile of the etched sample.

The process was monitored by taking photos of the etched samples and measuring profiles obtained using a profilometer. As seen in Figure 7 and Figure 8, the process results in the ingot-like shaping of the final integrated resonator, owing to the under-etching of the phosphoric acid below the Au coating of the sample. The surface shape is still acceptable despite some evidence of defects on the top due to minor turbulence created by the etching bath when used at 140 °C. On the other hand, especially when volume magnetostatic waves are excited in the resonator, the contribution of the surface to its resonance properties is still negligible, as demonstrated by the data in Figure 8, where the top of the sample is flat compared to the edge depth. The only change that might be considered in a full theory of the single and coupled resonators is the effective size of the individual resonator, accounting for the final shape after the etching.

The ingot shape due to the micromachining technique contributes to a wavevector spread and the necessity to introduce an averaged value of the wavevector along the thickness. Since the etching profile is linear, the intermediate value of the thickness could be a reasonable approximation to calculate the wavevector value, but an exact computation should be based on the following formula to calculate the average value of the excited wavevector:

(14) $$\langle k\rangle =\frac{1}{t}\int_{0}^{t}k\left(z\right)dz$$

Considering the dependence of k on the resonator size, i.e., k=nπ/l (when W≪l for rectangular resonators), we can elaborate the previous equation obtaining, for the first mode with *n* = 1, a dependence of the wavevector value from the quote *z*:

(15) $$k\left(z\right)=\frac{\pi }{l\left(z\right)}=\frac{\pi }{l\left(1-\frac{2}{\tan\alpha }\cdot \frac{z}{l}\right)}$$

where *z* is the quote ranging from 0 to *t* (thickness of the magnetic resonator), *l* is the bottom size of the ingot, and α is the etching angle between the inclined edge of the SER and its bottom. Using this definition, the Equation (14) is calculated as:

(16) $$\langle k\rangle =-\frac{\pi }{l}\cdot \frac{\ln\left(1-\frac{2}{\tan\alpha }\cdot \frac{t}{l}\right)}{\frac{2}{\tan\alpha }\cdot \frac{t}{l}}$$

The above calculation is important to conclude that an etched SER will have a wavevector spread, and, consequently, an intrinsic bandwidth enlargement. This does not involve additional losses like in the case of the magnetic full linewidth but can be properly tailored adjusting the etching angle depending on the used technology. Since an average value can be calculated, a spread is intrinsically expected and can be mathematically calculated even for a continuous variable.

**Figure 9:**
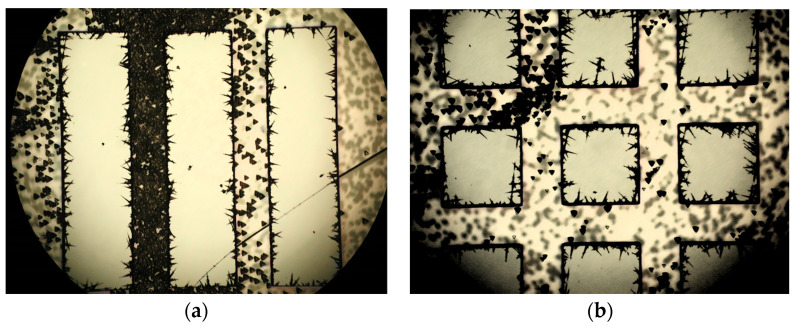
A detailed view of the etched structures is shown for (**a**) the rectangular resonators and (**b**) the matrix. The small triangular defects between the two resonators are due to the film’s and substrate’s crystallographic orientation, i.e., the <111>-direction.

The quality of the surface when the sample has been etched is clearly evidenced in Figure 9, using a polarized light microscope.

## 4. Experimental Results and Equivalent Circuits

In this section, we shall analyze the results of a ferromagnetic resonance (FMR) experiment on etched structures and the equivalent electrical circuits that are useful to model them. First, an FMR measurement has been performed on single resonators to derive the equivalent circuital components of the etched resonator. This is commonly practiced using the frequency of resonance, the bandwidth, and the quality factor. The single one has the exact nominal dimensions used for the coupled SERs obtained by the etching process, i.e., *l* × W = 3000 × 800 μm^2^. The experimental resonance frequency was set at 9.25 GHz, i.e., the frequency for the TE_102_ FMR cavity, with an externally applied DC magnetic field orthogonally placed to the film surface to obtain a full magnetic saturation in a direction normal to the sample. In these conditions, as from the classical literature about magnetostatic waves, the excitation of volume waves is obtained.

Then, the calculated circuital values for the resistance R, the inductance L, and the capacitance C were used to compare the FMR results obtained in a coupled SER structure with the circuital simulation.

The inductance is calculated using formulas of FMR based on the resonance frequency and the quality factor definition, from which we obtain *L_SER_* = 2160 nH, and the capacity is *C_SER_* = 1/*ω_res_*^2^*L_SER_*, where *ω_res_* = 2*πf_res_*. The resistance of the SER is obtained by its unloaded quality factor, resulting from the FMR measurement.

We have *R_SER_* = *ω_res_L_SER_*/*Q*_0_, while *Q*_0_ = *f_res_*/*γ*Δ*H*, where *γ* = 2.8 MHz/Oe is the gyromagnetic ratio, and Δ*H* is the magnetic full linewidth. In our case, it is Δ*H* ≈ 3 Oe, which means *Q*_0_ ≈ 1000 at 9.25 GHz. It turns out that *R_SER_* ≈ 125 ohm. The inferred values for the single resonator are helpful to obtain the *K*-values for circuital modeling of the coupled resonators. We can derive the *K*-coupling and estimate the response of a filter using resonators coupled with different separations, specifically λ/4, λ/2, and λ, meaning that λ/2 corresponds to the width of the resonator, with the exception performed for the necessary re-adjustment for the effective size due to both etching and boundary magnetic properties. We should also consider the possibility of including the external coupling factor *K_ext_*. The role played by *K_ext_* is that of the electrical matching with transducers or any microwave probe used for the excitation and the detection of the IN/OUT signals. In the case of FMR, we tuned the experimental conditions to obtain a critical coupling between the resonator and the cavity, but in practical applications, the kind of transducer to be used and the coupling degree must be considered for a correct prediction of the filter response.

**Figure 10:**
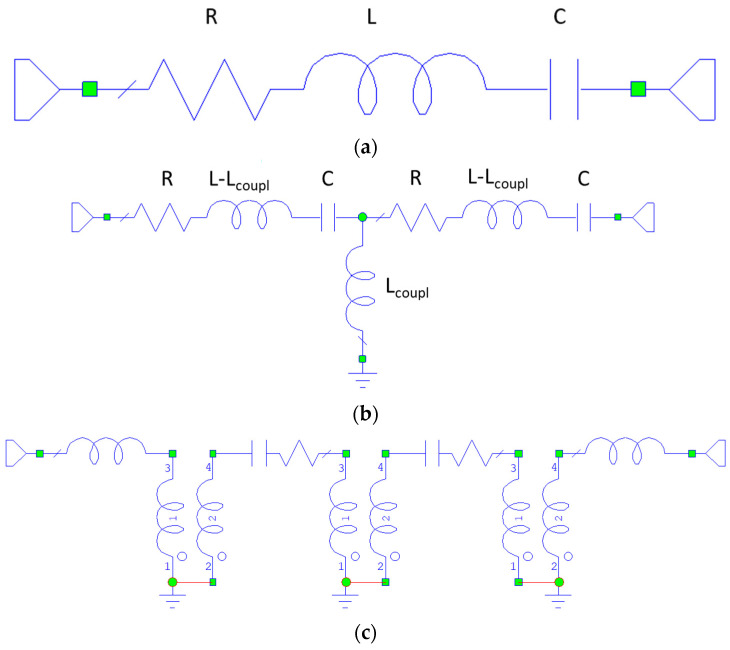
Equivalent circuits for the single resonator (**a**) with the simple series connection of the RLC components, and (**b**) two coupled ones with the insertion of a coupling capacitance L_coupl_ = KL. The magnetic nature of the resonators immediately suggests an inductive coupling. The inductive coupling is also evidenced in (**c**), where transformers can be alternatively used to model two series-connected resonators having an inductive coupling also with the I/O ports of the device. The indexes from 1 to 4 indicate the nodes in the electrical network.

**Figure 11:**
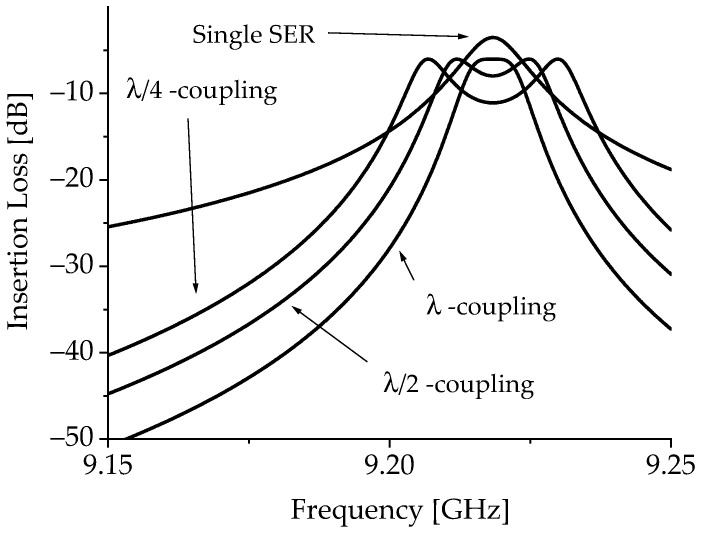
Simulation of the expected performance for a passband filter based on MSW resonators. The response of the single resonator based on the FMR data is compared with that of two coupled SERs, separated by λ (full wavelength), λ/2 (half wavelength), and λ/4 (quarter wavelength).

Figure 10 shows the equivalent circuit for the single resonator and the coupled ones. Additionally, a different arrangement can be considered for the coupled structure, with an explicit transformer between the two identical resonators, as expressed in Figure 10c. In Figure 11, the expected response of a two-port bandpass filter using coupled resonators is plotted based on the result of the resonance experiment.

The simulation of the following circuital structures has been performed using the above inferred values for the lumped components, including the linewidth, i.e., the losses, of the material.

The comparison between theory and experiment is constrained to the practical application of the proposed structure. Our aim was to demonstrate the potential application of the coupled configurations emphasizing the importance of appropriately designing and manufacturing microstrip transducers for effective coupling. Presently, we underline that the proposed structures are evaluated by means of FMR and their electrical response is used to simulate the potential application for an optimized transducer matching.

**Figure 12:**
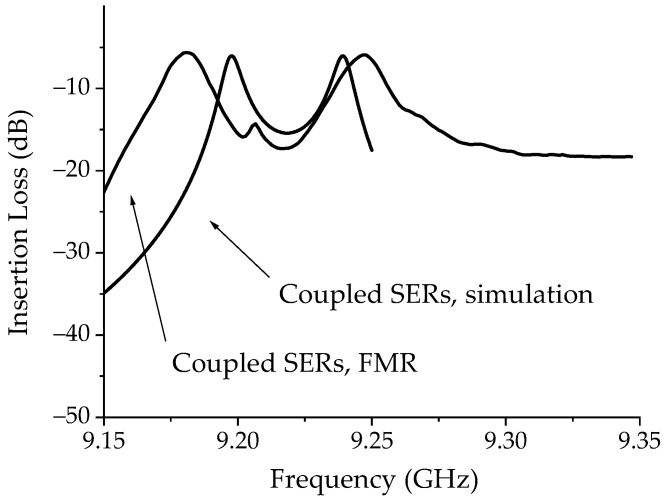
Comparison between the circuital simulation of two series coupled rectangular SERs and the FMR response.

The result for the λ/4 coupling is also shown in Figure 12, where the FMR data are compared to the simulation based on critical coupling. It is evident that the experimental response is not ideal, and the peaks originating from the coupling between resonators are larger than expected. As outlined in the previous discussion, there are different reasons, including the non-ideal geometry of the resonator edges after the etching.

**Figure 13:**
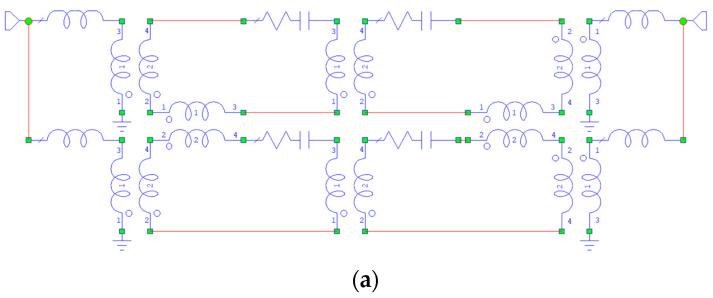

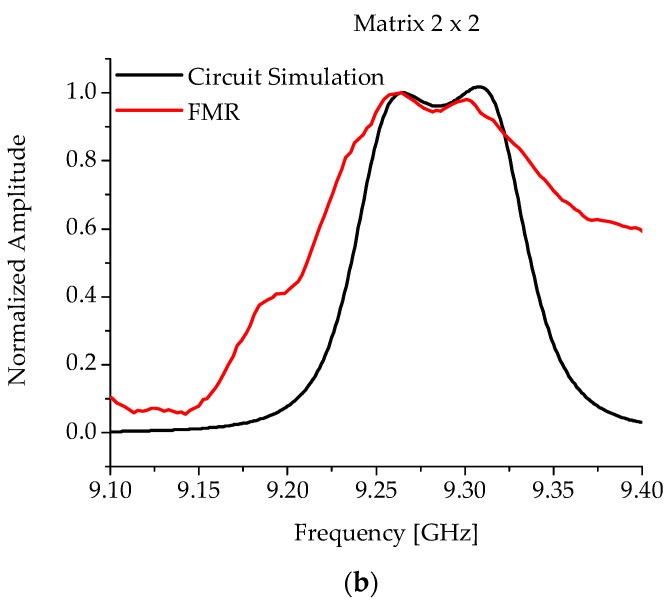
The circuit modeling in (**a**) has been used for the simulation in (**b**), compared to the FMR results from the same structure. Linear data have been normalized in both cases to the maximum to have a common scale useful for comparison.

In Figure 13a, the equivalent circuit used for the Matrix 2 × 2 simulation is shown, and in Figure 13b, the predicted performance of the Matrix is also shown compared to FMR data. The circuit has been simulated, accounting for equivalent transformers to model the coupling. This solution is more convenient owing to the Matrix shape and the position of the individual resonators, placed at a right angle or in series within the same structure.

From the analysis of data shown in Figure 13, it appears that a critical coupling with the external source, i.e., an electrical matching at the input/output ports of the Matrix, would help in reproducing the expected performance for the trailing and the leading edge of the filter response as a function of frequency. On the other hand, the position of the peaks has been correctly predicted and measured.

**Figure 14:**
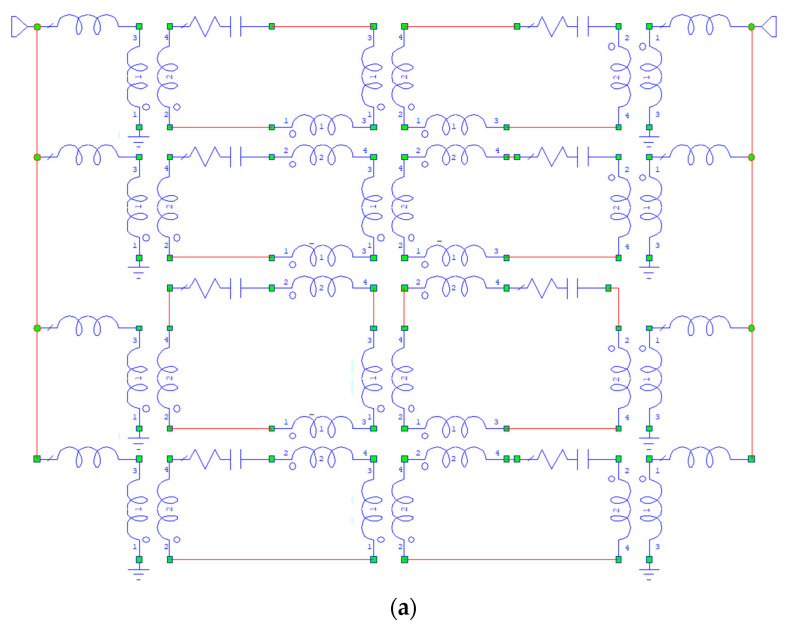

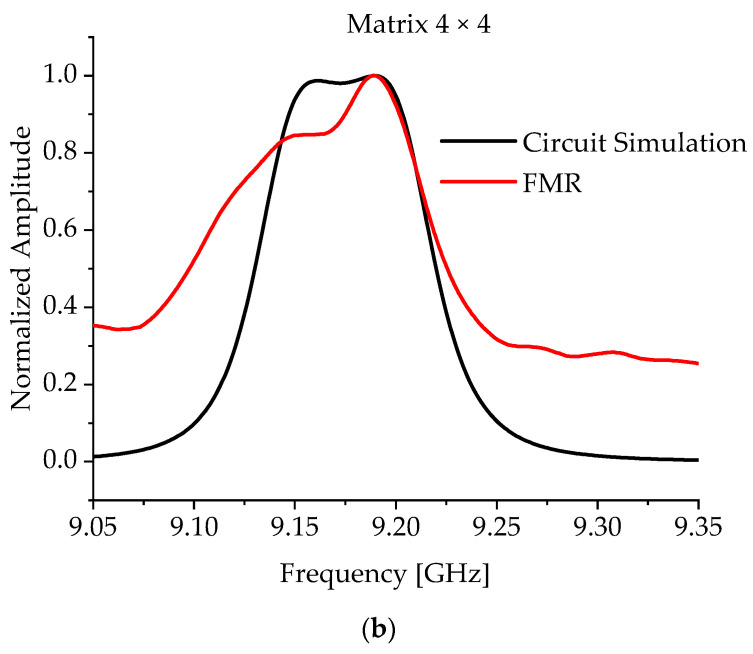
Equivalent circuit in (**a**) and electrical simulation compared to the FMR response for the Matrix 4 × 4 in (**b**). Data have been normalized to the maximum in linear scale.

In Figure 14, the same analysis has been performed for the Matrix 4 × 4, and both the equivalent circuit and the FMR response compared to the equivalent circuit simulation have been plotted.

The measured and simulated data presented in Figure 14 give evidence for the expected improvement in the sharpness of the filter response due to an increased number of coupled resonators. Again, like in the case of the 2 × 2 structure, the external quality factor of the FMR cavity did not guarantee an ideal electrical matching for the resonance experiment. On the other hand, a good input/output coupling provided by a microstrip transducer in an actual application, as it turns out from the circuit simulation obtained for a critical coupling of the structure to the external world, should result in a filter with the flat in-band response, and low insertion loss levels. Especially for the band-stop configurations, an SER top-coupled to a microstrip can be easily obtained to fulfill critical matching (50 ohms) conditions or different impedances if needed. The full modeling of the multilayer made by a grounded dielectric (typically alumina) and a microstrip loaded by a magnetic film epitaxially grown on a GGG substrate has been known since the 1970s, described in seminal papers like [42,43,44], reviews on applications [45], and further design developments from many authors after that [46,47,48,49,50]. Nanometer-thick films could also be excited by proper transducer design, but in these cases, the number of spins involved is lower because of a smaller volume, and the coupling efficiency limits the quality factor of a resonator to a few hundred units (Q ≈ 350) [51], while for notch filters and oscillators, Q-values exceeding 1000 are more desirable. Producing an efficient solution for magnetic film-based band-pass structures is more challenging because it requires a gap between the resonator and the transducer. On the other hand, the side coupling between a microstrip and a resonator is favored in the case of dielectric structures, using the electric field RF lines generated by the microstrip, while a top coupling favors the excitation of magnetic films using the RF magnetic field. The above considerations do not affect the design of a delay line because, in that case, a propagation mechanism is activated by using input/output transducers exciting the magnetic film like a waveguide, i.e., two microstrips with a sample as big as 1 cm in length and 0.5 cm in width (typical size), and propagation modes are transmitted along the path, naturally resulting in a band-pass response for bandwidths up to several hundred MHz. Extensive considerations can be found in [52] concerning the circuital simulation of resonators and their equivalent lumped components. More encouraging results for narrow-band filtering have been obtained in [53], where etched YIG thin films were produced with high quality factors in band-stop configurations using an ion milling technique for micromachining the magnetic film. A recent and interesting contribution parallel to our efforts can also be found in [54], where an etching technique with hot phosphoric acid at lower temperatures (80 °C) has been used to obtain miniaturized band-pass resonators. The above-referenced work in [53,54] would be a breakthrough for effectively implementing single and coupled MSW resonating structures in integrated configurations.

## 5. Conclusions

This paper proposes the detailed analytical and circuital modeling of a structure based on the technology of integrated coupled magnetostatic wave resonators. The approach represents an advancement from prior findings on individual resonators, mainly experimental, extended with a complete analytical model and further experimental findings using the FMR technique on the integrated, micromachined structures. The proposed circuital modeling is the natural output of this activity, aimed to demonstrate the possibility of using integrated structures to substitute bulk YIG spheres for multipole microwave filtering. From the circuital analysis, both series resonators and matrix-arranged SERs demonstrate a good perspective for microwave tunable filtering, providing an integrated solution characterized by reduced design efforts in the microwave feeding of the resonating structures. Such a configuration should be entirely considered in the plane of the microstrip circuitry, avoiding the need for complicated intricate 3D wire geometries to couple the resonators to the microwave field. In this way, only planar photolithographic processes should be needed. Further efforts should be dedicated to optimizing the shape of the etched resonators, thus improving not only the surface but also the resonator boundaries, i.e., the edges of the SERs, for a reliable prediction of the response of the etched structure. In principle, a microelectronic substrate like a high resistivity silicon (HRS), alumina, or GaAs, or even other advanced solutions like low-temperature cofired ceramics (LTCC) or gallium nitride (GaN) should be able to host the coupled MSW resonators integrated into a sub-system, because the ordinary photolithography pertains only to the substrate, while the etching is a separate process to obtain a structure suitable of a flip-chip assembly.

## Data Availability

The data presented in this study are available on request from the corresponding author.

## References

[1] Matthaei G.L., Young L., Jones E.M.T. (1985). Microwave Filters, Impedance-Matching Networks, and Coupling Structures.

[2] Zverev A.I. (1967). Handbook of Filter Synthesis.

[3] Helszajin J. (1967). YIG Resonators and Filters.

[4] Kabos P., Stalmakov V.S. (1994). Magnetostatic Waves and Their Applications.

[5] Prabhakar A., Stancil D.D. (2009). Spin Waves—Theory and Applications.

[6] Prabhakar A., Stancil D.D. (2021). Spin Waves—Problems and Solutions.

[7] Vittoria C. (1993). Microwave Properties of Magnetic Films.

[8] Cismaru A., Marcelli R. CPW Magnetostatic Wave Band Stop Resonators. Proceedings of the 2005 European Microwave Conference.

[9] Marcelli R., Nikitov S.A. (1996). Nonlinear Microwave Signal Processing: Towards a New Range of Devices. Nonlinear Microwave Magnetic and Magnetooptic Information Processing.

[10] Kalinikos B.A., Ustinov A.B., Wu M., Hoffmann A. (2013). Chapter Seven—Nonlinear Spin Waves in Magnetic Films and Structures: Physics and Devices. Solid State Physics.

[11] Sadovnikov A.V., Odintsov S.A., Beginin E.N., Grachev A.A., Gubanov V.A., Sheshukova S.E., Sharaevskii Y.P., Nikitov S.A. (2018). Nonlinear Spin Wave Effects in the System of Lateral Magnonic Structures. JETP Lett..

[12] Krivosik P., Patton C.E. (2010). Hamiltonian formulation of nonlinear spin-wave dynamics: Theory and applications. Phys. Rev. B.

[13] Wigen P.E. (1994). Nonlinear Phenomena and Chaos in Magnetic Materials.

[14] Rezende S.M. (2020). Fundamentals of Magnonics.

[15] AA. VV. (2022). Special Issue on Microwave Magnetics. Microw. Mag..

[16] Geiler M., Gillette S., Shukla M., Kulik P., Geiler A.L. (2021). Microwave Magnetics and Considerations for Systems Design. IEEE J. Microwaves.

[17] Bradley H., Louis S., Slavin A., Tyberkevych V. (2023). Pattern recognition using spiking antiferromagnetic neurons. arXiv.

[18] A Nikitov S., Kalyabin D.V., Lisenkov I.V., Slavin A., Barabanenkov Y.N., A Osokin S., Sadovnikov A.V., Beginin E.N., A Morozova M., A Filimonov Y. (2015). Magnonics: A new research area in spintronics and spin wave electronics. Physics-Uspekhi.

[19] Chumak A.V., Kabos P., Wu M., Abert C., Adelmann C., Adeyeye A.O., Akerman J., Aliev F.G., Anane A., Awad A. (2022). Advances in Magnetics Roadmap on Spin-Wave Computing. IEEE Trans. Magn..

[20] De Gasperis P., Marcelli R. (1987). A generalized ionic approach to the epitaxial growth of yttrium iron garnet films in molten solutions. Mater. Res. Bull..

[21] Rao Y.-H., Zhang H.-W., Yang Q.-H., Zhang D.-N., Jin L.-C., Ma B., Wu Y.-J. (2018). Liquid phase epitaxy magnetic garnet films and their applications. Chin. Phys. B.

[22] Tolksdorf W., Arend H., Hulliger J. (1989). Liquid Phase Epitaxy of Garnets. Crystal Growth in Science and Technology.

[23] Dubs C., Surzhenko O., Linke R., Danilewsky A., Brückner U., Dellith J. (2017). Sub-micrometer yttrium iron garnet LPE films with low ferromagnetic resonance losses. J. Phys. D Appl. Phys..

[24] Dubs C., Surzhenko O., Thomas R., Osten J., Schneider T., Lenz K., Grenzer J., Hübner R., Wendler E. (2020). Low damping and microstructural perfection of sub-40nm-thin yttrium iron garnet films grown by liquid phase epitaxy. Phys. Rev. Mater..

[25] Arsad A.Z., Zuhdi A.W.M., Ibrahim N.B., Hannan M.A. (2023). Recent Advances in Yttrium Iron Garnet Films: Methodologies, Characterization, Properties, Applications, and Bibliometric Analysis for Future Research Directions. Appl. Sci..

[26] Marcelli R., Rossi M., De Gasperis P. (1995). Coupled Magnetostatic Volume Wave Straight Edge Resonators for Multipole Microwave Filtering. IEEE Trans. Magn..

[27] Marcelli R., Koike T. (2005). Micromachined magnetostatic wave coupled resonators. IEEE Trans. Magn..

[28] Röschmann P. (1971). YIG filters. Philips Tech. Rev..

[29] Micro Lambda Wireless, Inc. Technology Description Yig Tuned Filters. https://www.microlambdawireless.com/resources/ytfdefinitions2.pdf.

[30] Linstrom B., Barnard J., Rogan J. (2022). Reinventing YIG Technology for Microwave Filter Applications. Microw. J..

[31] Krupka J., Salski B., Kopyt P., Gwarek W. (2016). Electrodynamic study of YIG filters and resonators. Sci. Rep..

[32] Popov M., Xiong Y., Zavislyak I., Chumak H., Fedorchuk O., Saha S., Bidthanapally R., Qu H., Page M.R., Srinivasan G. (2023). Y-type hexagonal ferrite-based band-pass filter with dual magnetic and electric field tunability. Sci. Rep..

[33] Micro Lambda Wireless, Inc. (2017). Yig-Based Closed Loop Signal Filtering and Amplitude Control. U.S. Patent.

[34] Pardavi-Horvath M., Yan J., Peverley J. (2001). Nonuniform internal field in planar ferrite elements. IEEE Trans. Magn..

[35] Pardo E., Chen D.-X., Sanchez A. (2004). Demagnetizing Factors for Square Bars. IEEE Trans. Magn..

[36] Lax B., Button J.K. (2012). Microwave Ferrites and Ferrimagnetics.

[37] Marcelli R., Sajin G., Cismaru A., Craciounoiu F. (2004). Band Stop Magnetostatic Waves Micromachined Resonators. Appl. Phys. Lett..

[38] Marcelli R., Sajin G., Cismaru A. (2004). Band-Pass Magnetostatic Wave Resonators on Micromachined Silicon Substrate. Rev. Sci. Instrum..

[39] TKoike T.K.T., Nakazawa H.N.H. (1994). Phase-Shift Control of Resonant Frequencies of Magnetostatic Wave Resonators. Jpn. J. Appl. Phys..

[40] Marcelli R., Rossi M., De Gasperis Su Jun P. (1996). Magnetostatic Wave Single and Multiple Stage Resonators. IEEE Trans. Magn..

[41] Heinrich B., Cochran J. (1993). Ultrathin metallic magnetic films: Magnetic anisotropies and exchange interactions. Adv. Phys..

[42] Ganguly A., Webb D. (1975). Microstrip Excitation of Magnetostatic Surface Waves: Theory and Experiment. IEEE Trans. Microw. Theory Tech..

[43] Ganguly A., Webb D., Banks C. (1978). Complex Radiation Impedance of Microstrip-Excited Magnetostatic-Interface Waves. IEEE Trans. Microw. Theory Tech..

[44] Sethares J. (1979). Magnetostatic Surface-Wave Transducers. IEEE Trans. Microw. Theory Tech..

[45] Ishak W. (1988). Magnetostatic wave technology: A review. Proc. IEEE.

[46] Freire M.J., Marques R., Medina F. A New Method for the Computation of the Insertion Loss of Magnetostatic-Surface Wave Transducers. Proceedings of the 2003 33rd European Microwave Conference.

[47] Freire M., Marques R., Medina F. (2003). Full-wave analysis of the excitation of magnetostatic-surface waves by a semi-infinite microstrip transducer—Theory and experiment. IEEE Trans. Microw. Theory Tech..

[48] Connelly D.A., Csaba G., Aquino H.R.O., Bernstein G.H., Orlov A., Porod W., Chisum J. (2021). Efficient electromagnetic transducers for spin-wave devices. Sci. Rep..

[49] Vanderveken F., Tyberkevych V., Talmelli G., Sorée B., Ciubotaru F., Adelmann C. (2022). Lumped circuit model for inductive antenna spin-wave transducers. Sci. Rep..

[50] Zagriadski S.V. (2002). Excitation and Reception of Electromagnetic, Magnetostatic and Spin Waves in Ferrite Films. Prog. Electromagn. Res..

[51] Costa J.D., Figeys B., Sun X., Van Hoovels N., Tilmans H.A.C., Ciubotaru F., Adelmann C. (2021). Compact tunable YIG-based RF resonators. Appl. Phys. Lett..

[52] Gao Q. (2021). An Equivalent Circuit Model for Tunable Bandpass Filters Based on Ferromagnetic Resonance. A Thesis Submitted in Partial Satisfaction of the Requirements for the Degree Master of Science in Electrical & Computer Engineering.

[53] Feng Y., Tiwari S., Bhave S.A., Wang R. (2023). Micromachined Tunable Magnetostatic Forward Volume Wave Bandstop Filter. IEEE Microw. Wirel. Technol. Lett..

[54] Devitt C., Wang R., Tiwari S. (2023). An Edge-Coupled Magnetostatic Bandpass Filter. arXiv.

